# Reduced fluoroquinolone susceptibility in Salmonella enterica serotypes in travelers returning from Southeast Asia.

**DOI:** 10.3201/eid0706.010613

**Published:** 2001

**Authors:** A Hakanen, P Kotilainen, P Huovinen, H Helenius, A Siitonen

**Affiliations:** Antimicrobial Research Laboratory, National Public Health Institute, Turku, Finland. antti.hakanen@utu.fi

## Abstract

During 1995 to 1999, we collected 1,210 Salmonella isolates; 629 were from Finnish travelers returning from abroad. These isolates were tested for susceptibility by determining MICs to ciprofloxacin, nalidixic acid, and seven additional antimicrobial agents. From 1995 to 1999, the annual proportion of reduced ciprofloxacin susceptibility (MIC > 0.125 microg/mL) among all travelers' isolates increased from 3.9% to 23.5% (p<0.001). The increasing trend was outstanding among the isolates from Southeast Asia; isolates from Thailand alone increased from 5.6% to 50.0% (p<0.001). The reduced fluoroquinolone susceptibility was nonclonal in character and significantly associated with multidrug resistance. A point mutation in the quinolone resistance-determining region of gyrA was present in all isolates with reduced susceptibility. These data provide further evidence for the rapid spread of multidrug-resistant pathogens from one continent to another.


### Reduced Fluoroquinolone Susceptibility in *Salmonella enterica* Serotypes in Travelers Returning from Southeast Asia

**Antti Hakanen,*† Pirkko Kotilainen,† Pentti Huovinen,* Hans Helenius,‡ and Anja Siitonen§**

*National Public Health Institute, Turku, Finland; †Turku University Central Hospital, Turku, Finland; ‡Turku University, Turku, Finland; and §National Public Health Institute, Helsinki, Finland

During 1995 to 1999, we collected 1,210 *Salmonella* isolates; 629 were from Finnish travelers returning from abroad. These isolates were tested for susceptibility by determining MICs to ciprofloxacin, nalidixic acid, and seven additional antimicrobial agents. From 1995 to 1999, the annual proportion of reduced ciprofloxacin susceptibility (MIC $\geq$ 0.125 µg/mL) among all travelers' isolates increased from 3.9% to 23.5% ($p<0.001$). The increasing trend was outstanding among the isolates from Southeast Asia; isolates from Thailand alone increased from 5.6% to 50.0% ($p<0.001$). The reduced fluoroquinolone susceptibility was nonclonal in character and significantly associated with multidrug resistance. A point mutation in the quinolone resistance-determining region of *gyrA* was present in all isolates with reduced susceptibility. These data provide further evidence for the rapid spread of multidrug-resistant pathogens from one continent to another.

Fluoroquinolones are active drugs against isolates of the *Salmonella* species (1). There are several reports, however, of treatment failures when these antimicrobials have been used to treat *Salmonella* infections caused by strains with reduced fluoroquinolone susceptibility (2-11). Some epidemiologic studies have shown that the number of *Salmonella* isolates with reduced fluoroquinolone susceptibility has increased, especially in Europe (12,13). Of particular note is the emergence of quinolone resistance in some clones of the widespread *Salmonella enterica* serotype Typhimurium definitive phage type 104 (hereafter *S.* Typhimurium DT104) (14,15). For example, Mølbak et al. (14) recently reported an outbreak caused by a quinolone-resistant *S.* Typhimurium DT104 clone that affected 27 patients in Denmark.

Our preliminary report on fluoroquinolone susceptibility of *Salmonella* isolates in Finland showed an increasing trend in quinolone resistance among isolates classified as being of foreign origin (16). Our study was performed to continue the survey of quinolone resistance and multidrug resistance among isolates of *S. enterica* serotypes. We collected and analyzed *Salmonella* isolates from Finnish patients who acquired the disease either at home or abroad during 1995 to 1999. Special attention was given to delineating the countries and areas associated with reduced fluoroquinolone susceptibility in salmonellae from travelers.

Address for correspondence: Antti Hakanen, Antimicrobial Research Laboratory, National Public Health Institute, P.O. Box 57, 20521 Turku, Finland; fax: 358-2-2519254; e-mail: antti.hakanen@utu.fi

### Methods

### *Salmonella* Isolates

In Finland, nearly all *Salmonella* isolates recovered from humans (annually 2,500 to 3,500) are sent to the National Salmonella Reference Centre of the National Public Health Institute for typing. In January 1995, a survey was started to monitor antimicrobial resistance in *Salmonella* isolates sent to the Reference Centre. During 1995 to 1999, we collected from this material a total of 1,210 *Salmonella* isolates, with the aim of including only epidemiologically unrelated strains. The possible relationship between different isolates was judged on the basis of epidemiologic information collected from the form that accompanied each isolate. Isolates recovered from distinct sources were determined to be epidemiologically unrelated.

*Salmonella* isolates were divided into two groups according to origin of infection. An isolate was designated to be from a traveler (i.e., foreign), if the patient had reported travel abroad during 1 month before the specimen date. All other isolates were designated to be of domestic (i.e., Finnish) origin. Isolates were collected in five phases: starting in January 1995, we consecutively collected 100 foreign and 100 domestic isolates; starting in September 1996, 200 foreign and 200 domestic isolates; and starting in January 1997, in January 1998, and in January 1999, 100 foreign and 100 domestic isolates, respectively.

### Susceptibility Testing

MICs of isolates were determined by the standard plate agar dilution method according to National Committee for Clinical Laboratory Standards (NCCLS) guidelines (17). The quinolones evaluated were ciprofloxacin and nalidixic acid; the other antimicrobials were ampicillin, cefotaxime, streptomycin, tetracycline, chloramphenicol, trimethoprim, and sulfamethoxazole. Mueller-Hinton II agar (BBL, Becton Dickinson and Co., Cockeysville, MD) was used as the culture media. *Staphylococcus aureus* American Type Culture Collection (ATCC) 29213, *Escherichia coli* ATCC 25922, *E.coli* ATCC 35218, and *Pseudomonas aeruginosa* ATCC 27853 were used as controls in testing for susceptibility.

The breakpoint value for reduced ciprofloxacin susceptibility was chosen as $\geq$0.125 µg/mL on the basis of earlier publications (14) and our recent scatterblot analyses, combined with the sequencing data of the quinolone resistance determining region (QRDR) of the *gyrA* gene (18). For other antimicrobials, MIC breakpoints for resistance used were those recommended by NCCLS (17).

Susceptibility data were analyzed by using the WHONET5 computer program (19,20), available from www.who.int/emc/WHONET/.

### Polymerase Chain Reaction and Sequencing

The QRDR of the *gyrA* gene was sequenced from isolates with reduced ciprofloxacin susceptibility, as we reported previously (18).

### Passenger Statistics

Data concerning the numbers of trips from Finland to countries of interest (i.e., countries with the largest numbers of all *Salmonella* isolates or isolates with reduced ciprofloxacin susceptibility) during the study months were received from Statistics Finland (www.stat.fi/).

### Statistical Analysis

Data were summarized with numbers and proportions of *Salmonella* isolates. Differences in these proportions were statistically tested by applying logistic regression analysis in the following way. Differences between years were modeled as a trend over years. Before doing these analyses, the goodness-of-fit of model of trend was tested. Differences between groups, in trends over years, were analyzed by testing interactions in the models. In addition to assessing crude differences between origin of isolates, an adjusted comparison with year as a covariate was done. Differences were quantified by calculating odds ratios and 95% confidence intervals (CI) (21). Differences between *Salmonella* infection rates in travelers returning from various travel destinations were statistically tested with Poisson regression analysis and quantified with infection rates and 95% CI (22); p values <0.05 were interpreted as significant. Statistical computing was performed by using the SAS system for Windows, release 8.00/1999 applying LOGISTIC or GENMOD procedures.

### Results

### Serotype and Origin of *Salmonella* Isolates

Of 1,210 *Salmonella* isolates studied, 629 were collected from persons reporting travel abroad within 1 month before specimen collection; 581 were classified as of domestic origin. Of all isolates, 98% were from stools and 2% were from extraintestinal sources. Ninety different serotypes were identified. Among the salmonellae isolated from travelers, *S.* Enteritidis and *S.* Typhimurium were the most prevalent serotypes, accounting for 36.2% and 7.8% of those isolates, respectively (Table 1). Among the domestic salmonellae, *S.* Typhimurium accounted for 37.3% and *S.* Enteritidis for 17.0% of isolates.

Of the 629 foreign isolates, the country where salmonellosis was acquired was identified for 618 isolates. For the remaining 11 isolates, origin could be traced to the continental level for 9; the origin of 2 isolates remains unknown.

Table 1. Serotype distribution and reduced ciprofloxacin (CIP) susceptibility (MIC $\geq$0.125 µg/mL) among 1,210 *Salmonella* isolates studied

| | Isolates from Finnish travelers | | Isolates of Finnish origin | | All isolates | |
|---|---|---|---|---|---|---|
| Serotype | No. of isolates (% of total) | % CIP MIC $\geq$0.125 µg/mL | No. of isolates (% of total) | % CIP MIC $\geq$0.125 µg/mL | No. of isolates (% of total) | % CIP MIC $\geq$0.125 µg/mL |
| *S.* Enteritidis | 228 (36.2) | 3.5 | 99 (17) | 0 | 327 (27) | 2.4 |
| *S.* Typhimurium | 49 (7.8) | 22.4 | 217 (37.3) | 1.4 | 266 (22) | 5.3 |
| *S.* Hadar | 34 (5.4) | 32.4 | 14 (2.4) | 21.4 | 48 (4) | 29.2 |
| *S.* Virchow | 27 (4.3) | 37.0 | 13 (2.2) | 15.4 | 40 (3.3) | 30.0 |
| *S.* Montevideo | 22 (3.5) | 0 | 15 (2.6) | 6.7 | 37 (3.1) | 2.7 |
| *S.* Newport | 18 (2.9) | 5.6 | 13 (2.2) | 0 | 31 (2.6) | 3.2 |
| *S.* Braenderup | 18 (2.9) | 0 | 2 (0.3) | 0 | 20 (1.7) | 0 |
| *S.* Infantis | 17 (2.7) | 5.9 | 33 (5.7) | 0 | 50 (4.1) | 2.0 |
| *S.* Anatum | 17 (2.7) | 11.8 | 4 (0.7) | 25.0 | 21 (1.7) | 14.3 |
| *S.* Panama | 11 (1.7) | 9.1 | 4 (0.7) | 0 | 15 (1.2) | 6.7 |
| Other serotypes (N=80) | 188 (29.9) | 11.2 | 167 (28.7) | 1.2 | 355 (29.3) | 6.5 |
| Total | 629 (100) | 10.5 | 581 (100) | 2.1 | 1,210 (100) | 6.4 |

Most isolates classified as foreign were from travelers to Asia and Europe (Table 2). Altogether, the isolates were obtained from travelers to 53 different countries, with Thailand, Spain, and Turkey the most frequent travel destinations (Table 3).

### Fluoroquinolone Susceptibility

Among all 1,210 *Salmonella* isolates, 78 (6.4%) exhibited reduced susceptibility to ciprofloxacin (MIC $\geq$0.125 µg/mL). These less susceptible isolates consisted of 66 isolates from travelers and 12 of domestic origin. From 1995 to 1999, the annual proportion of reduced ciprofloxacin susceptibility increased from 3.9% to 23.5% (p<0.001) among foreign isolates, and from 0% to 4.1% (p = 0.031) among domestic isolates (Figure, A,B). An increasing trend throughout the study period was confirmed by logistic regression analysis. The difference between isolates from travelers and those of domestic origin was significant, even after adjustment of year trends (p<0.001). The trends of these groups were not different (p = 0.684). All isolates with reduced ciprofloxacin susceptibility were uniformly resistant to nalidixic acid (MIC $\geq$32 µg/mL). Thus, the terms reduced fluoroquinolone susceptibility and quinolone resistance are used interchangeably hereafter. All these isolates were susceptible to ciprofloxacin according to NCCLS breakpoint recommendation (MIC $\leq$1 µg/mL).

The 78 isolates with reduced ciprofloxacin susceptibility included 19 different serotypes. The most common were *S.* Hadar (17.9% of isolates), *S.* Typhimurium (17.9%), *S.* Virchow (15.4%), and *S.* Enteritidis (10.3%).

### Quinolone Resistance in Travelers

Isolates with reduced ciprofloxacin susceptibility were obtained from travelers returning from 17 countries; most isolates were from Thailand, Israel, and Spain. The geographic distribution of these isolates by continent is shown in Table 2, which also presents the annual numbers of isolates with decreased ciprofloxacin susceptibility. During the study period, increases in quinolone-resistant isolates from Asia, taken as a whole, and from Southeast Asia alone, were statistically significant (p<0.001 for both). Among isolates from Thailand, this increase was especially prominent: from 1 (5.6%) of 18 in 1995 to 17 (50.0%) of 34 in 1999 (p<0.001) (Figure, C) (Table 4). Based on the estimated numbers of trips from Finland (during the time the isolates were collected) to the five most frequent countries of origin of all foreign *Salmonella* isolates, as well as of those with reduced ciprofloxacin susceptibility, the infection rates by quinolone-resistant *Salmonella* isolates were highest in travelers returning from Thailand and Malaysia: 0.81 and 0.80 infections per 1,000 trips, respectively (Table 5). (Although the total infection rate of salmonellosis was highest [6.7 infections per 1,000 trips] in Tunisia, the infection rate by quinolone-resistant *Salmonella* isolates was zero.) Despite the high proportion (58.3%) of reduced ciprofloxacin susceptibility in the 12 isolates from Israel (Table 3), the risk of acquiring quinolone-resistant salmonellosis was only 0.30 per 1,000 travels to that country (Table 5). Travelers returning from Spain and Estonia had low infection rates by all salmonellae, including the quinolone-resistant strains.

Among all 31 isolates with reduced ciprofloxacin susceptibility from Thailand, 13 different serotypes were identified; the 17 isolates collected during 1999 were divided into 12 serotypes (Table 4). These findings exclude the presence of one single clone or of a few clones.

Table 2. Number and source of 629 *Salmonella* isolates from Finnish travelers and the annual numbers of isolates with reduced ciprofloxacin susceptibility (MIC $\geq$0.125 µg/mL), 1995 to 1999

| | | Isolates with reduced ciprofloxacin susceptibility | | | | | | |
|---|---|---|---|---|---|---|---|---|
| | | Year[a] | | | | | | |
| Geographic area | All isolates | 1995 (102) | 1996 (216) | 1997 (107) | 1998 (102) | 1999 (102) | Total (% of all isolates) | p value[b] |
| Africa | **86** | **0** | **0** | **2** | **2** | **0** | **4 (4.7)** | **0.144** |
| America | **27** | **0** | **0** | **0** | **0** | **1** | **1 (3.7)** | **0.132** |
| Asia | **292** | **3** | **6** | **8** | **14** | **18** | **49 (16.8)** | **<0.001** |
| Southeast Asia | 147 | 1 | 1 | 6 | 10 | 18 | 36 (24.5) | <0.001 |
| Middle East | 93 | 2 | 5 | 1 | 2 | 0 | 10 (10.8) | 0.706 |
| Other areas | 52 | 0 | 0 | 1 | 2 | 0 | 3 (5.8) | 0.119 |
| Europe | **222** | **1** | **4** | **1** | **0** | **5** | **11 (5.0)** | **0.079** |
| Mediterranean area | 78 | 1 | 2 | 1 | 0 | 1 | 5 (6.4) | 0.801 |
| Canary Islands | 60 | 0 | 1 | 0 | 0 | 3 | 4 (6.7) | 0.147 |
| Other areas | 84 | 0 | 1 | 0 | 0 | 1 | 2 (2.4) | 0.205 |
| Total | 629[c,d] | 4[c] | 10[c] | 11[c] | 17[c,e] | 24[c] | 66[c,e] (10.5) | <0.001 |

[a] The annual numbers of all isolates from Finnish travelers studied in parentheses.
[b] The differences between years were modeled as a trend over years in these analyses. p value shows the statistical significance of this trend.
[c] Sum of the numbers written in bold.
[d] Two isolates of unknown origin included.
[e] One isolate of unknown origin included.

Table 3. List of the most frequent countries of origin of the 629 *Salmonella* isolates from Finnish travelers and percentage of reduced ciprofloxacin (CIP) susceptibility (MIC $\geq$0.125 µg/mL)

| Country/area | No. of isolates (% of total) | % CIP MIC $\geq$0.125 µg/mL |
|---|---|---|
| Thailand | 114 (18.1) | 27.2 |
| Spain (incl. Canary Islands) | 88 (14.0) | 6.8 |
| Turkey | 69 (11.0) | 4.3 |
| Tunisia | 37 (5.9) | 0 |
| Estonia | 32 (5.1) | 3.1 |
| Morocco | 27 (4.3) | 7.4 |
| India | 26 (4.1) | 7.7 |
| Greece | 17 (2.7) | 5.9 |
| Indonesia | 16 (2.5) | 6.3 |
| Cyprus | 14 (2.2) | 7.1 |
| Dominican Republic | 14 (2.2) | 7.1 |
| Kenya | 14 (2.2) | 7.1 |
| Sri Lanka | 14 (2.2) | 7.1 |
| Israel | 12 (1.9) | 58.3 |
| Malaysia | 11 (1.7) | 27.3 |
| Russia | 11 (1.7) | 0 |
| Other areas (N=42) | 113 (18.0) | 4.4 |
| Total | 629 (100) | 10.5 |

### Nucleotide Sequence Analysis

A base substitution in the QRDR of *gyrA* at codon 83 or 87 was present in all 78 isolates with reduced ciprofloxacin susceptibility. The sequence data of mutations in part of these isolates have been described elsewhere (18).

### Resistance to Other Antimicrobials

Among all 1,210 *Salmonella* isolates studied, 56 different resistance profiles were detected. These profiles were analyzed separately for the isolates with reduced ciprofloxacin susceptibility and those fully susceptible. As many as 47.4% of the 78 quinolone-resistant isolates had three or more additional resistance properties, whereas only 11.5% of the 1,132 quinolone-susceptible isolates were resistant to three or more antimicrobials ($p<0.001$).

Of all 629 foreign isolates and 581 domestic isolates, 20.3% and 18.1%, respectively, were resistant to tetracycline. Resistance to sulfamethoxazole was 14.0% among the foreign isolates and 17.7% among the domestic isolates. Corresponding figures were 7.9% and 11.5% for ampicillin and 5.6% and 5.3% for trimethoprim. Of all isolates, 16.9% were resistant to streptomycin and 8.8% were resistant to chloramphenicol. There was no resistance to cefotaxime.

### Discussion

Our study shows a dramatic increase in the annual proportion of reduced fluoroquinolone susceptibility (from 3.9% to 23.5%; $p<0.001$) among all foreign *Salmonella* isolates in Finland between 1995 and 1999. The increasing trend was particularly notable among isolates collected from travelers returning from Southeast Asia, especially Thailand. Moreover, 27.2% of all 114 *Salmonella* isolates from Thailand had reduced fluoroquinolone susceptibility; the proportion of reduced susceptibility was equal among the 11 isolates from Malaysia. The common nature of quinolone resistance in Southeast Asia is illustrated also by our passenger data, which revealed that a tourist's risk of acquiring quinolone-resistant salmonellosis was significantly higher in Thailand and Malaysia than in other destinations. These results clearly show that in the era of frequent international connections, microbes may be easily transmitted from one place to another. Correspondingly, factors furthering the emergence and spread of antimicrobial resistance in any country may soon have an impact on resistance of bacterial pathogens, or even of normal human flora, in faraway regions, even different continents. On this basis, the emergence of antimicrobial

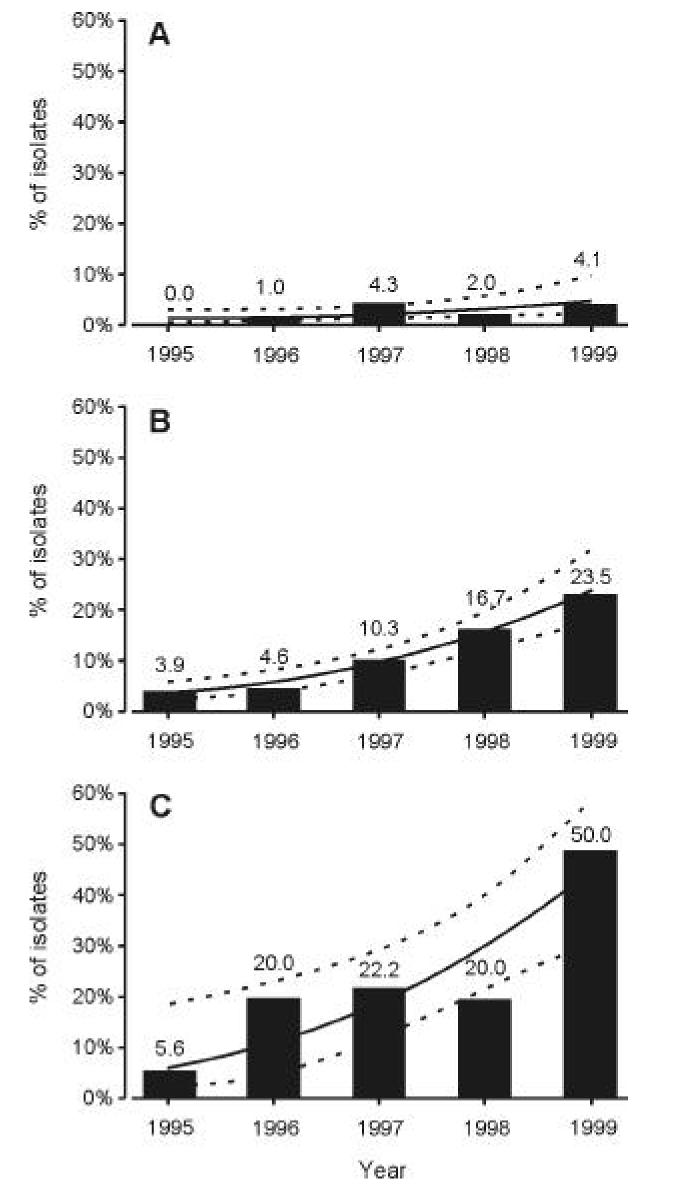

Figure. Percentage of *Salmonella* isolates with reduced ciprofloxacin susceptibility (MIC $\geq$0.125 µg/mL) of domestic (Finnish) origin (A), from Finnish travelers (B), and from Finnish travelers returning from Thailand (C), according to year. Bars represent observed percentages; the continuous curve represents the predicted trend of logistic model for the percentages; the dashed curves are 95% confidence intervals for the predictions.

Table 4. Serotype distribution of 31 *Salmonella* isolates with reduced ciprofloxacin susceptibility (MIC ≥0.125 µg/mL) from Thailand related to year of isolation

| | 1995 | 1996 | 1997 | 1998 | 1999 | Total |
|---|---|---|---|---|---|---|
| Serotype | (N=18/10)[a] | (N=5/4)[a] | (N=27/12)[a] | (N=30/17)[a] | (N=34/22)[a] | (N=114/31)[a] |
| *S.* Blockley | 1 | | | 1 | 2 | 4 |
| *S.* Haardt | | 1 | | 2[b] | 1 | 4 |
| *S.* Typhimurium | | | 6[b] | | | 6 |
| *S.* Rissen | | | | 2 | 1 | 3 |
| *S.* Hadar | | | | 1 | 3 | 4 |
| *S.* Enteritidis | | | | | 2 | 2 |
| *S.* Virchow | | | | | 2[b] | 2 |
| *S.* Albany | | | | | 1 | 1 |
| *S.* Anatum | | | | | 1 | 1 |
| *S.* Mbandaka | | | | | 1 | 1 |
| *S.* Newport | | | | | 1 | 1 |
| *S.* Panama | | | | | 1 | 1 |
| *S.* Schwarzengrund | | | | | 1 | 1 |
| Total | 1 | 1 | 6 | 6 | 17 | 31 |

[a] Number of all isolates/all serotypes.
[b] Among these isolates, two different resistance patterns were observed.

resistance in any part of the world may have a global bearing and thus deserves universal attention.

When looking for reasons for the rapidly increased quinolone resistance in our travelers' *Salmonella* isolates, three issues must be considered: transferable resistance, mutational resistance, and clonal spread. Until now, transferable resistance to the quinolone antimicrobial group has been described in one preliminary report (23). As far as we know, however, transferable fluoroquinolone resistance appears to be rare in bacteria in vivo. Thus, either clonal spread or resistance due to mutations in chromosomal genes remains the potential mechanism accounting for the high level of reduced fluoroquinolone susceptibility in Southeast Asia. In Thailand, the possibility of clonal spread as a major contributing factor was excluded by identification of 13 serotypes among the quinolone-resistant isolates. In addition, some of these serotypes contained different antimicrobial resistance patterns. Based on these data, we conclude that the reduced fluoroquinolone susceptibility of salmonellae in Thailand primarily involves mutations in chromosomal genes. This concept is consistent with our sequencing data: all 78 *Salmonella* isolates with reduced fluoroquinolone susceptibility (31 from Thailand) so far analyzed in our laboratory have shown a point mutation leading to an amino acid change in their QRDR of the *gyrA* gene. By no means does this finding exclude the presence of any other additional resistance mechanisms.

The emergence of mutation-based resistance may be fostered by selection pressure caused by the use of antimicrobial agents in either human medicine or agriculture. Accordingly, the alarming increase in quinolone resistance observed during the past few years among foodborne pathogens (24-27) has aroused speculation that this might be an effect of the use of quinolones in animal husbandry (14,28). Indeed, two recent articles (29,30) have shown that enrofloxacin (a fluoroquinolone used in agriculture) can select *Salmonella* mutants resistant to nalidixic acid and fluoroquinolones. No part of the world allows quinolones to be used as growth-promoters, but they have been licensed for therapeutic use in food animals in many countries. In Asia, several quinolones, including three fluoroquinolones licensed for humans (ciprofloxacin, ofloxacin, and norfloxacin), have been approved for animal use (31). In Europe, none of the fluoroquinolones licensed for humans are approved for animal use, although many other quinolone preparations are allowed for the treatment of livestock, poultry, and fish. The policy is more strict in the United States, where the only quinolone licensed for food animals is enrofloxacin (32), which is allowed for treatment of poultry alone (31). Without any data on the consumption figures of the quinolone antimicrobial group, no conclusions can be drawn on a potential link between the reduced fluoroquinolone susceptibility of salmonellae and the use of quinolones in animal husbandry in the areas studied. Yet, such a connection is plausible.

On the other hand, the alternative that extensive use of fluoroquinolones in human disease could be responsible for the rapidly increased quinolone resistance of salmonellae seems unlikely, since fluoroquinolones as potent bactericidal drugs are not particularly prone to select for resistance during treatment (33). In direct contrast, treatment with first-generation quinolones (e.g., nalidixic acid) is known to further rapid emergence of resistance in the family of *Enterobacteriaceae* (34,35). Consequently, widespread use of nalidixic acid could easily explain the emergence of reduced fluoroquinolone susceptibility in salmonellae. Again, in the absence of data on the potential use of nalidixic acid for treating salmonellosis in Southeast Asia, such an option can only be hypothesized. The theory is conceivable, however, considering that according to a recent report (36), nalidixic acid is frequently used in Thailand in the treatment of dysentery because resistance of shigella to other antimicrobial groups is common. Another topic of major interest involves

Table 5. Estimated travel-associated *Salmonella* infection rates in Finnish travelers

| Country | Est. no. of trips from Finland during study months[a] | No. of all isolates | Infection rate[b] | Rate ratio[c] | No. of isolates with CIP[d] MIC ≥0.125 µg/mL | Infection rate[b] by isolates with CIP MIC ≥0.125 µg/mL | Rate ratio[c] by isolates with CIP MIC ≥0.125 µg/mL |
|---|---|---|---|---|---|---|---|
| Thailand[e,f] | 38,180 | 114 | 3.0 | 1 | 31 | 0.81 | 1 |
| Spain (incl. Canary Islands)[e,f] | 391,310 | 88 | 0.2 | 0.08 (0.057-0.100) | 6 | 0.02 | 0.02 (0.008-0.045) |
| Turkey[e,f] | 45,427 | 69 | 1.5 | 0.51 (0.377-0.686) | 3 | 0.07 | 0.08 (0.025-0.266) |
| Tunisia[e] | 5,526 | 37 | 6.7 | 2.24 (1.548-3.249) | 0 | 0 | 0 (NA) |
| Estonia[e] | 135,128 | 32 | 0.2 | 0.08 (0.054-0.117) | 1 | 0.01 | 0.01 (0.001-0.067) |
| Israel[f] | 23,014 | 12 | 0.5 | 0.17 (0.096-0.317) | 7 | 0.30 | 0.37 (0.165-0.851) |
| Malaysia[f] | 3,747 | 11 | 2.9 | 0.98 (0.530-1.826) | 3 | 0.80 | 0.99 (0.302-3.225) |

[a]Based on the numbers of Finnish travelers to these countries; data collected from the reports of Statistics Finland.
[b]Infections per 1,000 trips.
[c]Thailand as the reference country. 95% confidence intervals in parentheses. The rate ratios between Thailand or Malaysia and other destinations were significant (p values <0.05).
[d]CIP = ciprofloxacin.
[e]Five most frequent countries of origin of all *Salmonella* isolates.
[f]Five most frequent countries of origin of *Salmonella* isolates with reduced ciprofloxacin susceptibility (MIC ≥0.125 µg/mL).

the potential influence of antimicrobial use in travelers for infections with quinolone-resistant *Salmonella* strains. Unfortunately, data on prophylactic or therapeutic use of antimicrobials were not collected here.

An increasing incidence of reduced fluoroquinolone susceptibility in *S. enterica* serotypes also became manifest in Europe during the 1990s. In England and Wales, reduced ciprofloxacin susceptibility (MIC ≥0.25 µg/mL) in salmonellae increased from 0.3% to 2.1% during the period 1991 to 1994, affecting primarily *S.* Hadar and *S.* Virchow serotypes (12). Concurrently, reduced ciprofloxacin susceptibility emerged in the multidrug-resistant clone of *S.* Typhimurium DT104, of which 1% in 1994 and 6% in 1995 were quinolone resistant (15). There are few U.S. reports of quinolone-resistant salmonellae. Only 21 (0.5%) of the 4,008 U.S. *Salmonella* isolates collected and analyzed during the years 1994 to 1995 were resistant to nalidixic acid (37). Even among the multidrug-resistant *S.* Typhimurium DT104, quinolone resistance in the United States has remained rare (24). However, strains with reduced fluoroquinolone susceptibility are currently not identified in any microbiologic laboratory worldwide according to current NCCLS recommendations, with MIC ≥4 µg/mL of ciprofloxacin as a breakpoint for resistance (17). These breakpoint values are considered adequate, as the clinical importance of the reduced fluoroquinolone susceptibility of salmonellae remains unproven. Nevertheless, we recommend that laboratories worldwide aim at recognizing these less susceptible strains, to reveal their eventual clinical impact. We suggest that laboratories use the nalidixic acid screening test (18) or the E-test to aid identification.

It is noteworthy that the less susceptible subpopulation has already undergone one point mutation and thus is potentially inclined to a second mutation, which could lead to high-level fluoroquinolone resistance. Admittedly, highly fluoroquinolone-resistant *Salmonella* strains are still extremely rare, and they are usually counterselected in field conditions (30). Even so, one can envision that highly fluoroquinolone-resistant *Salmonella* strains, capable of surviving, will inevitably emerge, if the less susceptible strains become prevalent and quinolone pressure persists. On this account, global surveillance of reduced fluoroquinolone susceptibility of salmonellae is also necessary for epidemiologic reasons.

Simultaneous with the increasing incidence of quinolone resistance in salmonellae, rapid emergence of fluoroquinolone resistance is occurring in other enteric bacteria, especially *Campylobacter* sp. and *E. coli* (25-27,38), a situation that threatens to impede the effectiveness of this antimicrobial group. The significantly more common multidrug resistance observed here among the quinolone-resistant salmonellae compared with the susceptible population (47.4% vs. 11.5%) is also of concern. This finding suggests that the use of fluoroquinolones may select for multidrug resistance among salmonellae and provokes a question of whether the same could happen among other bacterial species. In that case, the likelihood of the emergence of notable pathogens, resistant to fluoroquinolones as well as to other commonly used drugs, will certainly increase if the consumption of fluoroquinolones continues to grow. Collectively, these data indicate that prudent use of the quinolone antimicrobial group is warranted to prevent further development of resistance and to preserve the usefulness of these valuable drugs.

In conclusion, we have shown a dramatic increase in reduced fluoroquinolone susceptibility in salmonellae from travelers returning from Southeast Asia. The reduced susceptibility of salmonellae to the fluoroquinolone group was significantly associated with multidrug resistance. Moreover, all quinolone-resistant *Salmonella* isolates had undergone a point mutation in the QRDR of the *gyrA* gene. In contrast to previous reports on quinolone resistance in a specific clone or in a few *Salmonella* serotypes, the reduced fluoroquinolone susceptibility of our isolates was nonclonal. These data provide further evidence of the rapid spread of multidrug-resistant pathogens from one continent to another. The emergence of antimicrobial resistance in any part of the world may have global implications and is, therefore, of universal concern.

### Acknowledgments

We thank Ritva Marin for providing the data on passenger statistics; Liisa Immonen, Minna Lamppu, Tuula Randell, and all the staff members at the laboratories of the study for expert technical assistance; and Maarit Green for language revision.

Supported by grants from the Maud Kuistila Memorial Foundation, the Research Foundation of Orion Corporation, and Turku University Central Hospital (special EVO government grant), all to Dr. Hakanen.

Dr. Hakanen is a research physician in the Antimicrobial Research Laboratory, National Public Health Institute, Turku, Finland. His research interest is focused on antimicrobial resistance of enteric pathogens.

### References

1. Asperilla MO, Smego RA Jr, Scott LK. Quinolone antibiotics in the treatment of *Salmonella* infections. Rev Infect Dis 1990;12:873-89.
2. Brown JC, Thomson CJ, Amyes SG. Mutations of the *gyrA* gene of clinical isolates of *Salmonella typhimurium* and three other *Salmonella* species leading to decreased susceptibilities to 4-quinolone drugs. J Antimicrob Chemother 1996;37:351-6.
3. Launay O, Van J-CN, Buu-Hoi A, Acar JF. Typhoid fever due to a *Salmonella typhi* strain of reduced susceptibility to fluoroquinolones. Clin Microbiol Infect 1997;3:541-3.
4. Le Lostec Z, Fegueux S, Jouve P, Cheron M, Mornet P, Boisivon A. Reduced susceptibility to quinolones in *Salmonella typhi* acquired in Europe: a clinical failure of treatment. Clin Microbiol Infect 1997;3:576-7.
5. McCarron B, Love WC. Acalculous nontyphoidal salmonellal cholecystitis requiring surgical intervention despite ciprofloxacin therapy: report of three cases. Clin Infect Dis 1997;24:707-9.
6. Ouabdesselam S, Tankovic J, Soussy CJ. Quinolone resistance mutations in the *gyrA* gene of clinical isolates of *Salmonella*. Microbiol Drug Resist 1996;2:299-302.
7. Pers C, Søgaard P, Pallesen L. Selection of multiple resistance in *Salmonella enteritidis* during treatment with ciprofloxacin. Scand J Infect Dis 1996;28:529-31.
8. Piddock LJ, Griggs DJ, Hall MC, Jin YF. Ciprofloxacin resistance in clinical isolates of *Salmonella typhimurium* obtained from two patients. Antimicrob Agents Chemother 1993;37:662-6.
9. Rowe B, Ward LR, Threlfall EJ. Ciprofloxacin-resistant *Salmonella typhi* in the UK. Lancet 1995;346:1302.
10. Vasallo FJ, Martin-Rabadan P, Alcala L, Garcia-Lechuz JM, Rodriguez-Creixems M, Bouza E. Failure of ciprofloxacin therapy for invasive nontyphoidal salmonellosis. Clin Infect Dis 1998;26:535-6.
11. Wain J, Hoa NT, Chinh NT, Vinh H, Everett MJ, Diep TS, et al. Quinolone-resistant *Salmonella typhi* in Viet Nam: molecular basis of resistance and clinical response to treatment. Clin Infect Dis 1997;25:1404-10.
12. Frost JA, Kelleher A, Rowe B. Increasing ciprofloxacin resistance in salmonellas in England and Wales 1991-1994. J Antimicrob Chemother 1996;37:85-91.
13. Piddock LJ, Ricci V, McLaren I, Griggs DJ. Role of mutation in the *gyrA* and *parC* genes of nalidixic-acid-resistant *Salmonella* serotypes isolated from animals in the United Kingdom. J Antimicrob Chemother 1998;41:635-41.
14. Mølbak K, Baggesen DL, Aarestrup FM, Ebbesen JM, Engberg J, Frydendahl K, et al. An outbreak of multidrug-resistant, quinolone-resistant *Salmonella enterica* serotype typhimurium DT104. N Engl J Med 1999;341:1420-5.
15. Threlfall EJ, Frost JA, Ward LR, Rowe B. Increasing spectrum of resistance in multiresistant *Salmonella typhimurium*. Lancet 1996;347:1053-4.
16. Hakanen A, Siitonen A, Kotilainen P, Huovinen P. Increasing fluoroquinolone resistance in salmonella serotypes in Finland during 1995-1997. J Antimicrob Chemother 1999;43:145-8.
17. National Committee for Clinical Laboratory Standards; Performance standards for antimicrobial susceptibility testing: ninth informational supplement. Vol. 19, No 1. Wayne (PA): The Committee; 1999. (NCCLS document no. M-100-S9.)
18. Hakanen A, Kotilainen P, Jalava J, Siitonen A, Huovinen P. Detection of decreased fluoroquinolone susceptibility in salmonellas and validation of nalidixic acid screening test. J Clin Microbiol 1999;37:3572-7.
19. O'Brien TF, Stelling JM. WHONET: an information system for monitoring antimicrobial resistance. Emerg Infect Dis 1995;1:66.
20. Stelling JM, O'Brien TF. Surveillance of antimicrobial resistance: the WHONET program. Clin Infect Dis 1997;24 Suppl 1:S157-68.
21. Agresti A. Categorial data analysis. New York: John Wiley & Sons; 1990.
22. McGullagh P, Nelder JA. Generalized linear models. London: Chapman and Hall; 1989.
23. Martínez-Martínez L, Pascual A, Jacoby GA. Quinolone resistance from a transferable plasmid. Lancet 1998;351:797-9.
24. Glynn MK, Bopp C, Dewitt W, Dabney P, Mokhtar M, Angulo FJ. Emergence of multidrug-resistant *Salmonella enterica* serotype typhimurium DT104 infections in the United States. N Engl J Med 1998;338:1333-8.
25. Smith KE, Besser JM, Hedberg CW, Leano FT, Bender JB, Wicklund JH, et al. Quinolone-resistant *Campylobacter jejuni* infections in Minnesota, 1992-1998. N Engl J Med 1999;340:1525-32.
26. Sáenz Y, Zarazaga M, Lantero M, Gastañares MJ, Baquero F, Torres C. Antibiotic resistance in *Campylobacter* strains isolated from animals, foods, and humans in Spain in 1997-1998. Antimicrob Agents Chemother 2000;44:267-71.
27. Prats G, Mirelis B, Llovet T, Muñoz C, Miró E, Navarro F. Antibiotic resistance trends in enteropathogenic bacteria isolated in 1985-1987 and 1995-1998 in Barcelona. Antimicrob Agents Chemother 2000;44:1140-5.
28. Levy SB. Multidrug resistance—a sign of the times. N Engl J Med 1998;338:1376-8.
29. Medders WM, Wooley RE, Gibbs PS, Shotts EB, Brown J. Mutation rate of avian intestinal coliform bacteria when pressured with fluoroquinolones. Avian Dis 1998;42:146-53.
30. Giraud E, Brisabois A, Martel JL, Chaslus-Dancla E. Comparative studies of mutations in animal isolates and experimental in vitro- and in vivo-selected mutants of *Salmonella* spp. suggest a counterselection of highly fluoroquinolone-resistant strains in the field. Antimicrob Agents Chemother 1999;43:2131-7.
31. Division of Emerging and Other Communicable Disease Surveillance and Control. Use of quinolones in food animals and potential impact on human health: report of a WHO meeting: Geneva, Switzerland; 1998 Jun 2-5. Geneva: World Health Organization; 1998. (Document no. WHO/EMC/ZDI/98.10.)
32. McKellar Q, Gibson I, Monteiro A, Bregante M. Pharmacokinetics of enrofloxacin and danofloxacin in plasma, inflammatory exudate, and bronchial secretions of calves following subcutaneous administration. Antimicrob Agents Chemother 1999;43:1988-92.

33. Hooper DC, Wolfson JS. Fluoroquinolone antimicrobial agents. N Engl J Med 1991;324:384-94.
34. Ronald AR, Turck M, Petersdorf RG. A critical evaluation of nalidixic acid in urinary-tract infections. N Engl J Med 1966;275:1081-9.
35. D'Alessio DJ, Olexy VM, Jackson GG. Oxolinic acid treatment of urinary-tract infections. Antimicrob Agents Chemother 1967;7:490-6.
36. Hoge CW, Bodhidatta L, Tungtaem C, Echeverria P. Emergence of nalidixic acid resistant *Shigella dysenteriae* type 1 in Thailand: an outbreak associated with consumption of a coconut milk dessert. Int J Epidemiol 1995;24:1228-32.
37. Herikstad H, Hayes P, Mokhtar M, Fracaro ML, Threlfall EJ, Angulo FJ. Emerging quinolone-resistant *Salmonella* in the United States. Emerg Infect Dis 1997;3:371-2.
38. Garau J, Xercavins M, Rodríguez-Carballeira M, Gómez-Vera JR, Coll I, Vidal D, et al. Emergence and dissemination of quinolone-resistant *Escherichia coli* in the community. Antimicrob Agents Chemother 1999;43:2736-41.
